# The gut commensal *Faecalibacterium hominis* attenuates indole—AhR signaling and restores ASD—like behaviors with BTBR mice

**DOI:** 10.3389/fmicb.2025.1640149

**Published:** 2025-08-22

**Authors:** You Yu, Yujing Wang, Jie Zhang, Shucheng Li, Yulin Wang, Xin You, Xue Chen, Mengxuan Du, Lisheng Xie, Shuang-Jiang Liu

**Affiliations:** ^1^State Key Laboratory of Microbial Diversity and Innovative Utilization, Institute of Microbiology, Chinese Academy of Sciences, Beijing, China; ^2^State Key Laboratory of Biopharmaceutical Preparation and Delivery, Chinese Academy of Sciences, Beijing, China; ^3^State Key Laboratory of Microbial Technology, Shandong University, Qingdao, China; ^4^Department of Rheumatology and Clinical Immunology, Peking Union Medical College Hospital, Chinese Academy of Medica Sciences & Peking Union Medical College, Beijing, China

**Keywords:** the gut-brain axis, autism spectrum disorders (ASD), microbiome, indole, aryl hydrocarbon receptor (AhR), BTBR

## Abstract

Autism spectrum disorders (ASD), a group of neurodevelopmental disorders characterized by the core symptoms of impaired social communication and stereotyped behaviors, is strongly associated with dysregulated microbiota-gut-brain axis. Emerging evidence suggests that *Faecalibacterium*, which showed reduced abundance in ASD cohorts, holds therapeutic potential, though its interaction with host remain unexplored. Here, we investigated the efficacy and molecular basis of *Faecalibacterium hominis* 4P-15 (4P-15) in BTBR *T*^+^ *Itpr3*^*tf*^/J (BTBR) mice, an idiopathic ASD mouse model. Oral administration of 4P-15 significantly reduced the intestinal levels of indole, indole-3-propionic acid (IPA), and indole-3-acetic acid (IAA), as well as the level of IPA in brain. Furthermore, the decreased levels of IPA in brain contributed to the attenuated aryl hydrocarbon receptor (AhR) signaling characterized by increased expression of downstream elements, including glutamate transporters and GABA receptors. Ultimately, this modulation led to the restoration of excitatory/inhibitory imbalance, a typical pathophysiological feature of ASD, and thereby alleviated ASD core behavioral symptoms. Our findings underscore *Faecalibacterium*-mediated AhR modulation as a promising therapeutic strategy for ASD, highlighting the dual potential of *Faecalibacterium*-based probiotics and targeted interventions against indole-AhR signaling to address neurodevelopmental disorders.

## Introduction

Autism spectrum disorders (ASD), a group of neurodevelopmental disorders characterized by core symptoms of impaired social communication (e.g., reduced social reciprocity and atypical eye contact) and stereotyped behaviors such as stereotypical motor movements and insistence on sameness, has increasingly been linked to dysregulated gut-brain axis (Association, 2013). Emerging evidence highlights the gut microbiome as a pivotal modulator of neurodevelopment, with microbiota dysbiosis now recognized as a hallmark of ASD. Studies consistently report altered gut microbial composition in ASD patients and animal models (Liu et al., 2019; Lou et al., 2022; Chen et al., 2019). Notably, microbial signatures in ASD significantly correlate with the core symptoms, underlining the potential clinical applicability of the fecal microbiome for aiding in the diagnosis of ASD (Su et al., 2024; Wan et al., 2024; Manghi et al., 2024). Furthermore, therapeutic strategies targeting the gut microbiome, such as fecal microbiota transplantation, probiotic colonization, and prebiotic and dietary interventions, have shown promise in alleviating ASD symptoms (Li et al., 2024; Sgritta et al., 2019; Kang et al., 2017; Yu et al., 2025). These advances position gut microbiota remodeling as a viable therapeutic frontier in ASD.

Emerging evidence highlights the gut microbiome as a pivotal regulator of neurodevelopment by modulating synaptic plasticity and neurotransmitter synthesis, primarily via microbial metabolites, immune activation, and vagal nerve signaling. In recent years, bacteria-derived metabolites have garnered significant attention as key mediators in the microbiota-gut-brain axis, influencing how commensal gut microbes shape host neural activity. Indole and its derivatives, produced through the metabolism of tryptophan by gut microbes, have emerged as crucial elements influencing neurological function (Agus et al., 2018; Zhou et al., 2023). Cohort studies have indicated a correlation between indoles and abnormalities in the nervous system. Elevated levels of several indoles, such as indole, indole-3-acetic acid, and 3-methylindole, have been observed in the feces and urine of children with ASD (Khan et al., 2022), suggesting the potentially detrimental effect of indole and its derivatives. As the receptor for indole and its derivatives, the aryl hydrocarbon receptor (AhR) plays a critical role in regulating the transcription of various downstream genes, some of which are responsible for neurotransmission. Furthermore, AhR signaling pathway is dysregulated in the brain of ASD mice. However, the mechanisms through which gut microbiota impact the AhR signaling pathway in ASD and how this interference affects ASD-related phenotypes via the gut-brain axis remain poorly understood.

*Faecalibacterium*, emerging as a next-generation probiotic candidate, has drawn significant interest due to its anti-inflammatory properties and ability to enhance intestinal barrier integrity (Sokol et al., 2008; Lapiere et al., 2020). Notably, a reduced abundance of *Faecalibacterium* has been observed in multiple cohorts of individuals with ASD (Kang et al., 2018; Zhao et al., 2023; Pang et al., 2023; De Angelis et al., 2013; Ding et al., 2020; Wan et al., 2022) and the abundance of *Faecalibacterium* was negatively associated with the severity of ASD (Ding et al., 2020), yet its therapeutic potential and underlying mechanisms in ASD remain largely unexplored. In this study, we focused on *Faecalibacterium hominis* 4P-15 (4P-15), a new species isolated by our team from the feces of a typically developing individual (Liu et al., 2021), to assess its efficacy and investigate its underlying mechanisms using the BTBR *T*^+^ *Itpr3*^*tf*^/J (BTBR) mice, a widely recognized idiopathic model of ASD. The BTBR mice exhibit pronounced social deficits, as evidenced by their avoidance of social interaction with unfamiliar mice in three-chamber social interaction test and stereotypic behaviors supported by excessive self-grooming in self-grooming test. Our investigation identifies that 4P-15 normalizes abnormal levels of indole and its derivatives in the intestine and brain, and corrects the downstream of AhR signaling pathway associated with excitatory/inhibitory imbalance in ASD, thereby alleviating core behavioral deficits of ASD. Our investigation underscores the significance of intestinal commensal bacteria in regulating ASD and highlights potential mechanisms, laying a theoretical groundwork for probiotic intervention and indicating a fresh perspective for the clinical management of ASD by targeting indole and its derivatives and AhR signaling.

## Results

### *Faecalibacterium hominis* 4P-15 has been isolated and cultured from fecal samples of the typically developing individual

**Figure 1:**
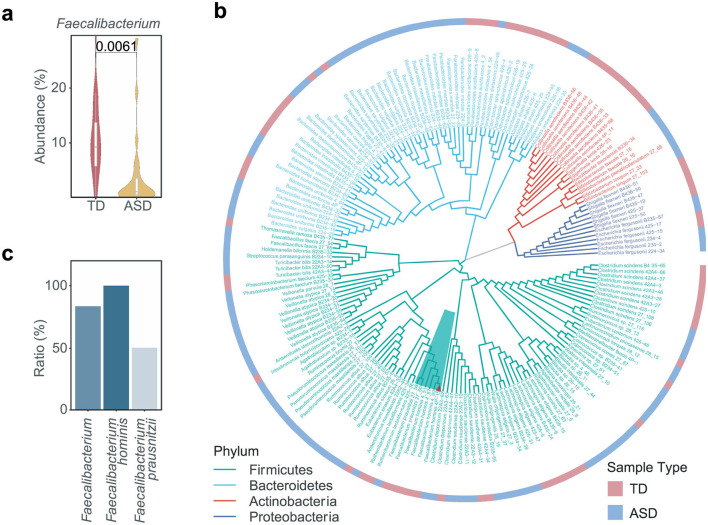
The isolation and culture of *Faecalibacterium* from fecal samples. **(a)** The relative abundance of *Faecalibacterium* between TD and ASD individuals was shown. The figure was based on the original data of a selected ASD cohort (ID number 11169 in the database “Qiita”) (Kang et al., 2018). TD, typically developing children. **(b)** The phylogenetic tree depicts the isolated gut microbial strains from TD and ASD samples. The branches are color-coded to represent four distinct phyla, with the dark green background specifically highlighting the genus *Faecalibacterium*. The red triangle denotes the strain *Faecalibacterium hominis* 4P-15. The heatmap surrounding the phylogenetic tree indicates the type of sample from which each strain was isolated. **(c)** The ratio of *Faecalibacterium* strains isolated from fecal samples of the TD individual compared to those isolated from fecal samples of both TD and ASD individuals. Quantitative data are shown as the mean ± SEM **(a)**. The *P*-value was determined by the two-sided Wilcoxon rank-sum test and the adjusted *P*-value was evaluated using the false discovery rate (FDR) correction of *P*-value **(a)**, and the significance was indicated by adjusted *P*-value **(a)**.

We observed that the abundance of *Faecalibacterium* reduced in ASD individuals compared to typically developing (TD) individuals in several cohorts (Kang et al., 2018; Zhao et al., 2023; Pang et al., 2023; De Angelis et al., 2013; Ding et al., 2020), suggesting that *Faecalibacterium* may play a protective role in the context of ASD (Figure 1a and Supplementary Figure 1a). To obtain the microbial strains belonging to *Faecalibacterium*, we isolated and cultured the gut microbes derived from the fecal samples of TD and ASD individuals. We harvested 183 bacterial strains belonging to 65 species (Figure 1b). Focusing on *Faecalibacterium*, two *Faecalibacterium prausnitzii* strains and four *Faecalibacterium hominis* strains were obtained (Figure 1b). Notably, *F. hominis* was identified as a new species, and all strains of this species were isolated from the TD sample (Figure 1c). Consequently, we selected the strain *F. hominis* 4P-15 (4P-15) for further functional research (Figure 1b, the red triangle).

### 4P-15 has shown potential in alleviating the core deficits of ASD in BTBR mice

**Figure 2:**
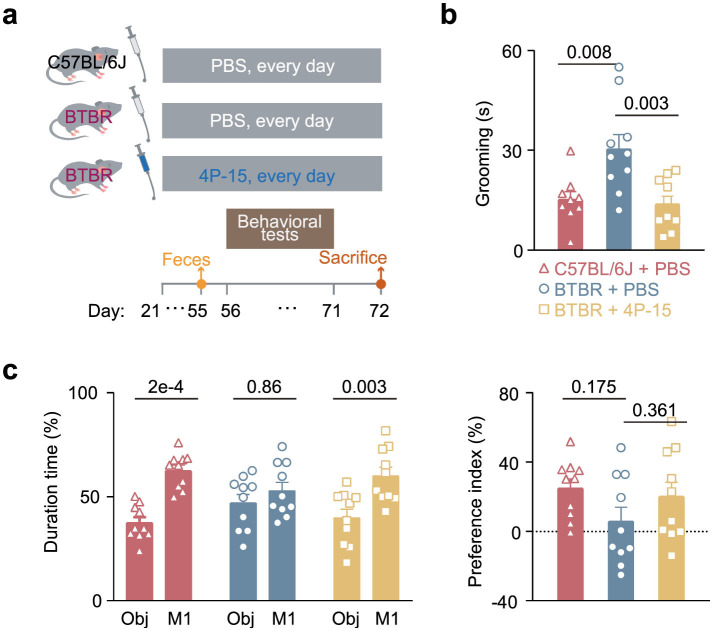
The restoration of core ASD-like behaviors in BTBR mice by *F. hominis* 4P-15. **(a)** Schematic diagram of the study design. **(b)** Grooming time in the self-grooming test. *n* = 10 mice for each group. **c**, Percentage of duration time (left panel) and the preference index (right panel) in the social ability session of the three-chamber social interaction test. Ob, object. *n* = 10 mice for each group. Quantitative data are shown as the mean ± SEM. The adjusted *P*-values were determined by one-way ANOVA with two-tailed Tukey's multiple comparison tests [**(b)** and **(c)** (right panel)] and two-way ANOVA with two-tailed Turkey's test for multiple comparisons (**c**, left panel). Significance was indicated by adjusted *P*-values **(b, c)**.

To explore whether 4P-15 could play a protective role in ASD by alleviating the ASD-like phenotypes in ASD animal model, we gavaged weaned BTBR mice daily for 4 weeks (Figure 2a). Subsequently, we evaluated ASD-relevant behaviors using the three-chamber test for social interaction deficits and the self-grooming test for stereotyped behavior. The results indicated that 4P-15 restored the stereotyped behavior in the self-grooming test (Figure 2b) and the impaired social interaction in the social ability session of the three-chamber test (Figure 2c and Supplementary Figure 2a), the core deficits of ASD. Furthermore, we conducted the open-field test for anxiety and the sucrose preference test for depression to assess the impact of 4P-15 on these behaviors. We did not observe any significant improvement of 4P-15 on anxiety or depression (Supplementary Figures 2b, c). Furthermore, we analyzed the correlation between the abundance of *F. hominis* in the feces and behavioral outcomes, and found a significant negative correlation between *F. hominis* abundance and stereotypic behavior in the *F. hominis*-gavaged BTBR mice (Supplementary Figures 2d, e).

### 4P-15 does not affect inflammatory cytokines and SCFAs in the intestine and neurotransmitters in the brain

**Figure 3:**
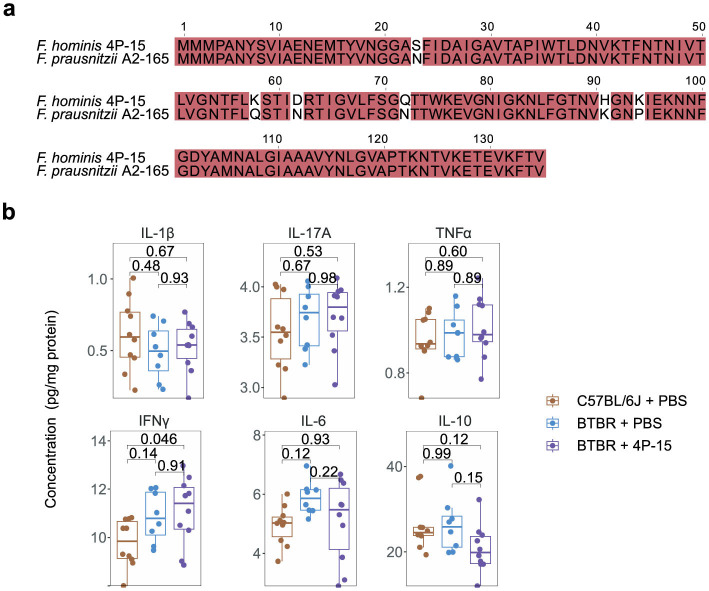
The non-effect of *F. hominis* 4P-15 on inflammatory cytokines, neurotransmitters, or SCFAs in BTBR mice. **(a)** The microbial anti-inflammation molecule (MAM) amino acid sequence alignment between *F. hominis* 4P-15 and *Faecalibacterium prausnitzii* A2-165. The identical sequences were highlighted in red. **(b)** The concentrations of inflammatory cytokines in the cerebral cortex of the brain. *n* = 10, 8 and 10 mice, respectively. Quantitative data are shown as the mean ± SEM. The adjusted *P*-values were determined by one-way ANOVA with two-tailed Tukey's multiple comparison tests **(b)**. Significance was indicated by adjusted *P*-values **(b)**.

Next, we explored the alterations associated with the mechanisms of the gut-brain axis in both the intestine and the brain in response to 4P-15 administration (Yu, 2021). Given that the genome of 4P-15 contained a microbial anti-inflammatory molecule showing 96.56% similarity (https://blast.ncbi.nlm.nih.gov/Blast.cgi) to *F. prausnitzii* (Auger et al., 2022) (Figure 3a), we hypothesized the anti-inflammatory properties of 4P-15 and the anti-inflammatory effect might mediate the protective effect of 4P-15 on ASD-like phenotypes. We analyzed six inflammatory cytokines in the cerebral cortex following gavage and found that 4P-15 did not affect the levels of these cytokines (Figure 3b). Then, we examined bacteria-derived compounds such as SCFAs and neurotransmitters that were previously reported to have altered levels in BTBR mice and participate in the modulation of CNS activity by gut microbes (Liu et al., 2024; Bove et al., 2024). Our results showed no significant changes in the levels of SCFAs in the feces or neurotransmitters in the brain after 4P-15 treatment (Supplementary Figures 2f, g).

### 4P-15 restores the abnormal levels of intestinal indole and its derivatives by modulating the potential indole-producing bacteria

**Figure 4:**
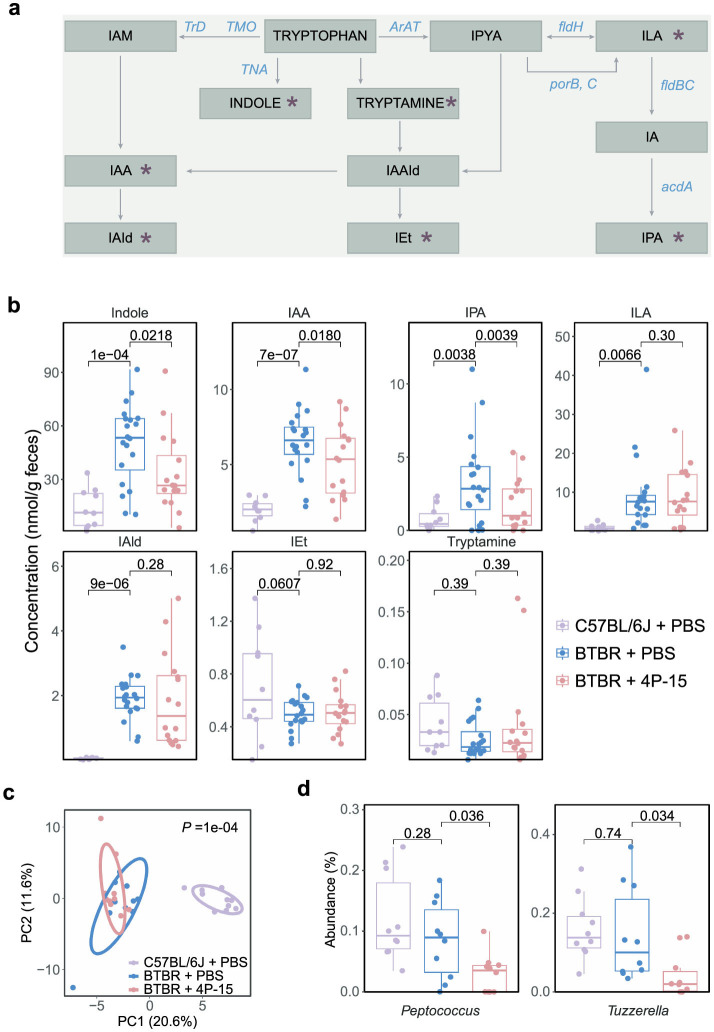
The modulation of intestinal indole and its derivatives, as well as gut microbes by *F. hominis* 4P-15 in BTBR mice. **(a)** The metabolites and enzymes in the indole pathway of tryptophan metabolism. The metabolites analyzed in this study were marked by red asterisks. IAM, indole-3-acetamide. IAA, indole-3-acetic acid. IAId, indole-3-aldehyde. IAAId, indole-3-acetaldehyde. IEt, indole-3-ethanol. IPYA, indole-3-pyruvate. ILA, indole-3-lactic acid. IPA, indole-3-propionic acid. TrD, tryptophan decarboxylase. TMO, tryptophan 2-monooxygenase. TNA, tryptophanase. ArAT, aromatic amino acid aminotransferase. fldH, phenylacetate dehydrogenase. porB, C, pyruvate: ferredoxin oxidoreductase B and C. fldBC, phenylacetate dehydratase. acdA, acyl-CoA dehydrogenase. **(b)** Box plots demonstrating the concentrations of indole and its six derivatives in the feces of C57BL/6J mice gavaged with PBS, BTBR mice gavaged with PBS, and BTBR mice gavaged with 4P-15. *n* = 10, 20, 16 mice, respectively. The adjusted *P*-values were calculated using one-way ANOVA with multiple comparisons by controlling FDR. The significant difference was defined as the adjusted *P*-value < 0.05. **(c, d)** A 16S rRNA sequencing analysis of gut microbiota from C57BL/6J mice treated with PBS, BTBR mice treated with PBS, and BTBR mice treated with 4P-15. *n* = 10 mice for each group. **(c)** PCA plots of the fecal microbiota composition at the genus level (PERMANOVA). **(d)** The relative abundance of genera significantly different between BTBR treated with 4P-15 and PBS. The significantly different genera were defined as the adjusted *P*-value < 0.05, and the adjusted *P*-values were evaluated using the FDR correction of *P*-value (one-way ANOVA). Quantitative data are shown as the mean ± SEM **(b, d)**. Significance was indicated by adjusted *P*-values **(b, d)**.

Next, we explore the functional mechanisms via the gut-brain axis following the 4P-15 intervention, focusing on the intrinsic defects of the ASD mouse model we utilized. Previous studies reported excessive activation of the AhR signaling pathway in BTBR mice, characterized by increased expression of AhR (Uddin et al., 2023). Hyperactivation of AhR may lead to neurodevelopmental toxicity, contributing to the pathogenesis of ASD (Kimura et al., 2017; Siddiqui et al., 2021; Martin et al., 2022). In our investigation, we sought to determine whether 4P-15 could alleviate the hyperactivation of AhR by reducing the levels of AhR ligands, such as indole and its derivatives, which are primarily produced by gut bacteria and function mainly through AhR activation. We analyzed levels of indole and six indole derivatives in fecal samples (Figure 4a). Our results revealed elevated levels of indole and four derivatives, indole-3-acetic acid (IAA), indole-3-propionic acid (IPA), indole-3-lactic acid (ILA) and indole-3-aldehyde (IAId) in the feces of BTBR mice compared to C57BL/6J mice (Figure 4b). Notably, administration of 4P-15 led to a reduction in the levels of indole, IPA, and IAA (Figure 4b).

Subsequently, we delved into understanding how the administration of 4P-15 reduces the levels of intestinal indole and its derivatives. We first examined whether the genome of 4P-15 contained the enzymes capable of indole biotransformation and did not identify any functional elements (Ma et al., 2018) (Supplementary Figure 3a). We then assessed whether 4P-15 had the potential to decrease the concentrations of indole, indole-3-acetic acid (IAA), and indole-3-propionic acid (IPA) *in vitro*. By comparing the concentrations of these three compounds in the fermentation broth samples of 4P-15 compared with its YCFA medium, we observed no significant difference in IAA levels (Supplementary Figure 3b). Since the concentrations of indole and IPA were below the detection threshold in YCFA medium, we supplemented the YCFA medium with indole and IPA, respectively. Our findings indicated that 4P-15 failed to reduce the concentrations of both indole and IPA in the supplemented YCFA medium (Supplementary Figures 3c, d).

Next, our focus shifted to the influence of 4P-15 on the gut microbiota, particularly the abundance of indole-producing bacteria. Utilizing 16S rRNA gene sequencing of cecal contents, we found that 4P-15 administration did not significantly alter either alpha or beta diversity (Figure 4c and Supplementary Figures 3e–h). Subsequent analysis revealed a significant reduction in the abundance of two genera following 4P-15 treatment (Figure 4d). Notably, one of these genera, *Peptococcus*, is identified as a potential indole-producing bacterium (Lanigan, 1976; Schwan, 1979). These results suggest that 4P-15 might decrease intestinal indole concentrations by reducing the abundance of potential indole-producing bacteria.

### 4P-15 restores the abnormal levels of indole derivatives and AhR downstream pathways related to ASD pathology in BTBR mice

**Figure 5:**
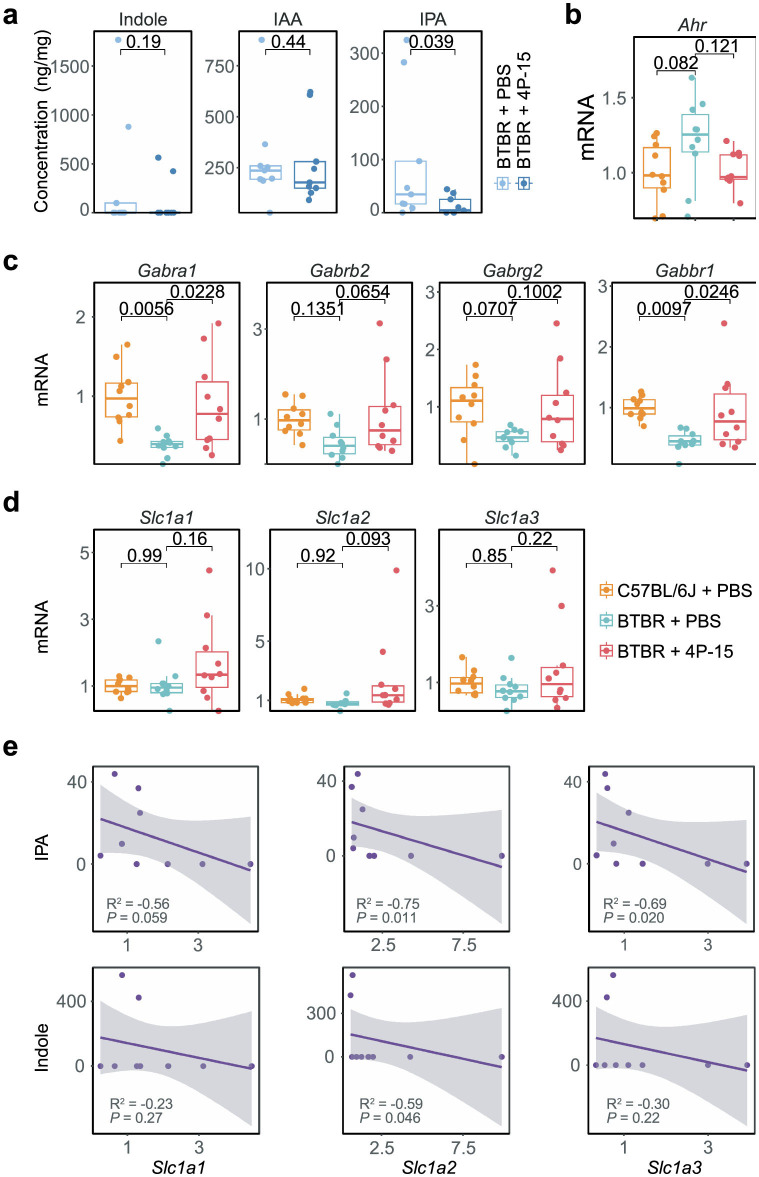
The modulation of indoles and AhR signaling by *F. hominis* 4P-15 in the brain of BTBR mice. **(a)** Box plots showing the concentration of indole, IAA, and IPA in the cerebellum of BTBR mice gavaged with PBS and 4P-15. *n* = 9 mice for each group. The *P*-values were calculated by the one-sided Student's *t*-test. The significant difference was defined as the *P*-value < 0.05. **(b–d)** Box plots showing the qPCR analysis in the cerebral cortex of C57BL/6J mice gavaged with PBS, BTBR mice with PBS, and BTBR mice with 4P-15. *n* = 10 mice for each group. The adjusted *P*-values were calculated using one-way ANOVA with Tukey's multiple comparisons test. The significant difference was defined as the adjusted *P*-value < 0.1. **(e)** Scatter plots showing the association between the mRNA levels of AhR downstream genes (*X*-axis) and the concentrations of AhR ligands (*Y*-axis) in BTBR mice gavaged with 4P-15. *n* = 8 mice for each group. Statistical significances (*P*-value) and correlation coefficients (*R*^2^) were determined by the function *cor.test* (method = “spearman”) in R. The significant difference was defined as the *P*-value < 0.05. Quantitative data are shown as the mean ± SEM **(a–d)**. Significance was indicated by *P*-value **(a, e)** and adjusted *P*-value **(b–d)**.

We next examined whether 4P-15 gavage-induced reductions in intestinal indole and its derivatives influenced brain metabolite concentrations. 4P-15 administration significantly decreased IPA levels and modestly reduced indole concentrations in the brain (Figure 5a). To assess the potential neuroprotective effects of these metabolic changes, we prioritized AhR-regulated pathways directly relevant to ASD pathophysiology. While AhR modulates numerous downstream targets, we focused on glutamate transporters (*Slc1a1, Slc1a2*, and *Slc1a3*) and GABA receptors (*Gabra1, Gabrb2, Gabrg2*, and *Gabbr1*), given their dual rationale: (1) prior studies demonstrate AhR suppresses glutamate transporter expression (Silva-Parra et al., 2024) and GABA receptor transcription (Dever et al., 2016), and (2) ASD pathogenesis involves excitatory/inhibitory imbalance driven by glutamate-GABA signaling dysregulation. While 4P-15 administration did not significantly change *Ahr* mRNA level (Figure 5b and Supplementary Figure 4a), it notably upregulated the expression of glutamate transporters *Slc1a2* and GABA receptors *Gabra1, Gabrb2*, and *Gabbr1* (Figures 5c, d)

To investigate how AhR ligand dynamics influence its downstream targets, specifically glutamate transporters and GABA receptors, we analyzed correlations between brain indole/IPA levels and AhR pathway gene expression in BTBR mice gavaged with 4P-15. Notably, IPA concentrations exhibited significant inverse associations with *Slc1a2*/*Slc1a3* expression, while indole levels showed a significant negative correlation specifically with *Slc1a2* (Figure 5e and Supplementary Figure 4b). Together, these data suggest that 4P-15 derepresses glutamate transporters and GABA receptors through diminished AhR signaling by reducing indole-related AhR ligands, thereby alleviating excitatory-inhibitory imbalance.

## Discussion

**Figure 6:**
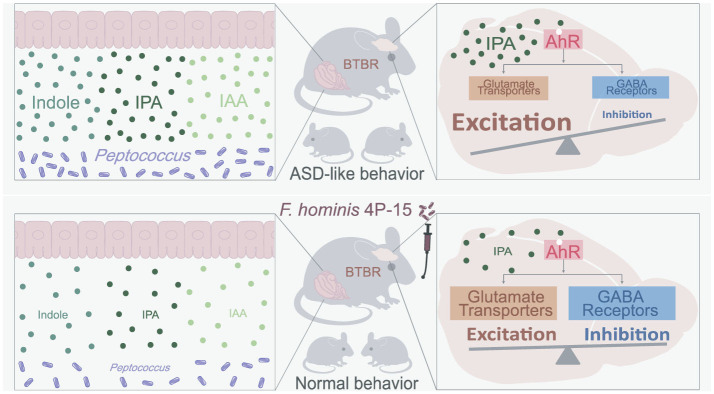
The summary of how 4P-15 alleviates the ASD-like phenotypes in BTBR mice via the gut-brain axis. Oral administration of 4P-15 attenuated the concentrations of intestinal indole and its derivatives IPA and IAA in BTBR mice possibly by reducing the abundance of *Peptococcus*, potential indole-producing bacteria. Furthermore, 4P-15 decreased the levels of indoles, especially IPA in the brain and increased the expression of glutamate transporter and GABA receptors, which were downstream elements of the AhR signaling and were negatively regulated by it. Ultimately, this led to the restoration of excitation/inhibition imbalance and ASD-like behaviors.

Our study identifies *Faecalibacterium hominis* 4P-15 as a pioneering microbial therapeutic avenue in ameliorating ASD pathophysiology in BTBR mice through gut-brain axis modulation. By reducing indole and its derivatives in both the gut and brain, 4P-15 rectifies dysregulated AhR signaling pathways linked to excitatory-inhibitory imbalance in ASD, and thus ameliorates core behavioral deficits (Figure 6). These findings establish a new therapeutic paradigm for ASD by targeting indole-AhR signaling pathways, offering clinically translatable strategies.

Our prior work demonstrated that *Bacteroides uniformis* ameliorates ASD-associated excitatory/inhibitory imbalance in CHD8 haploinsufficient mice by attenuating intestinal amino acid transport, thereby decreasing glutamate precursor availability for excitatory neurotransmission (Yu et al., 2022b; Ji et al., 2025). Intriguingly, while both *B. uniformis* and *F. hominis* 4P-15 alleviate ASD-like behaviors, they operate through distinct yet complementary mechanisms: 4P-15 reduces microbial indoles to suppress AhR-driven suppression of glutamate/GABA synaptic transmission regulators, whereas *B. uniformis* directly reduces glutamate synthesis via amino acid metabolic rewiring. This mechanistic divergence underscores the potential for personalized microbiota-based therapies tailored to individual ASD subpopulations. For instance, 4P-15 may preferentially benefit patients with elevated fecal indole levels and AhR overactivation, whereas *B. uniformis* could target those exhibiting dysregulated amino acid metabolism. Such stratification aligns with the growing recognition of ASD as a disorder of heterogeneous etiology, where microbiome-directed interventions must align with host-specific metabolic signatures to optimize efficacy.

Our research demonstrates that 4P-15 decreases the expression of genes downstream of the indole-AhR signaling pathway, which is linked to the regulation of excitatory-inhibitory imbalance in ASD. While prior investigations into AhR's role in ASD have predominantly focused on its canonical activation of cytochrome P450 enzymes and associated oxidative stress and inflammation pathways (Dhulkifle et al., 2021; Ahmed et al., 2022), the connection between microbial AhR ligands and synaptic excitatory-inhibitory dysregulation has remained unexplored. Notably, we demonstrate that 4P-15 administration reduces bacterial-derived neuroactive metabolites, particularly indole and its derivatives, which are potent AhR agonists. This reduction attenuates AhR overactivation, resulting in increased expression of glutamate transporters and GABA receptors, critical regulators of neurotransmission equilibrium. By shifting the focus from AhR's traditional metabolic roles to its synaptic regulatory functions, our findings provide distinctive mechanistic insights into how microbial modulation of the indole-AhR axis may rectify excitatory-inhibitory imbalance in ASD.

Our findings suggest that indole and its derivatives, including IPA and IAA, may exert neurotoxic effects in ASD, as evidenced by their association with AhR overactivation and disrupted excitatory-inhibitory balance. However, this contrasts with reports in other neurological contexts where indole derivatives exhibit neuroprotective properties (Hwang et al., 2009; Serger et al., 2022; Yin et al., 2023). These divergent roles may stem from context-dependent factors such as metabolite concentration, tissue-specific AhR activation thresholds, or disease-specific neuroinflammatory microenvironments. In ASD, where chronic neuroinflammation and glutamate excitotoxicity are hallmarks, indole derivatives might act as double-edged swords: at physiological levels, they could maintain gut-brain homeostasis, but their pathological accumulation, as observed in our study, may tip the balance toward synaptic dysfunction. Resolving this paradox will require delineating dose-dependent effects, AhR isoform specificity, and interactions with comorbid factors in future studies.

Our study revealed that oral administration of 4P-15 reduced the abundance of *Peptococcus* in the gut microbiota, though the precise inhibitory mechanisms remain uncharacterized. Interbacterial suppression within microbial communities commonly involves resource competition, metabolite-mediated inhibition, immune modulation, or niche occupation (Jiang et al., 2025; Ikryannikova et al., 2020). Carbon source utilization analysis demonstrated distinct substrate preferences between the *Faecalibacterium* and *Peptococcus*: *Faecalibacterium* primarily metabolizes fermentable sugars (e.g., fructose, glucose, lactose) (Liou et al., 2024; Sakamoto et al., 2022), whereas *Peptococcus* specializes in proteolytic metabolism, relying on protein-derived substrates such as peptones, amino acids, and polypeptides (Reece et al., 1976; Wilkins et al., 1975; Shkoporov et al., 2016). This divergence in nutritional niches suggests limited direct competition for carbon sources under typical dietary conditions. Instead, metabolite-mediated inhibition emerged as a plausible mechanism. *Faecalibacterium* generates multiple organic acids, including SCFAs (e.g., butyrate), lactate, and formate (Sakamoto et al., 2022), which collectively lower intestinal luminal pH. This pH reduction likely creates a hostile microenvironment that selectively inhibits the growth of pH-sensitive *Peptococcus* (Lanigan, 1976).

While our study highlights the role of 4P-15 in alleviating ASD-like behaviors through the indole-AhR-glutamate/GABA axis, additional mechanisms may contribute to its neuromodulatory effects. First, although we observed reduced indole levels in the brain and prior studies suggest indoles can cross the blood-brain barrier (Guo et al., 2023; Pappolla et al., 2021; Kunevičius et al., 2024), we cannot exclude indirect effects mediated by peripheral AhR activation. Intestinal epithelia and immune cells express AhR (Madison et al., 2023; Rothhammer and Quintana, 2019), whose activation may modulate gut-brain communication via skewed T cell differentiation or intestinal barrier function (Hao and Whitelaw, 2013; Stockinger et al., 2021). Second, the upregulation of glutamate transporters and GABA receptors by 4P-15 may involve AhR-independent pathways. For instance, indole and its derivatives can act as agonists or antagonists of serotonin receptors and tropomyosin receptor kinases (TrKs) (Tammiku-Taul et al., 2016; van Niel et al., 1999; Bermudez et al., 1990; Swain et al., 1991; Hogendorf et al., 2019), which regulate synaptic plasticity and are implicated in neurodevelopmental disorders (Stoleru et al., 2013). Additionally, indole and its derivatives are predicted to interact with GABA_A_ receptor-associated proteins, potentially enhancing receptor surface expression (Chen et al., 2005; Leil et al., 2004). These putative mechanisms underscore the pleiotropic nature of microbial metabolites in neuroregulation and warrant further investigation.

While our study primarily focused on male BTBR mice to investigate the effects and underlying mechanisms of 4P-15, we acknowledge that clinical data demonstrate a depletion of *Faecalibacterium* in both male (Kang et al., 2018) and female ASD cohorts (PRJEB42687). This raises the possibility that females with ASD could also benefit from the 4P-15 intervention. However, it remains uncertain whether the underlying mechanisms, particularly indole-AhR modulation, operate similarly across sexes. Emerging evidence highlights significant sex differences in the regulation of the gut-brain axis. It is plausible that hormonal-immune crosstalk may contribute to differential responses to microbiota interventions between males and females. Therefore, while we believe that the core mechanism of 4P-15 is likely applicable to both sexes, it is crucial to validate these findings in sex-stratified ASD models to identify sex-specific nuances. Future research in this area could clarify the necessity for personalized treatment regimens, such as hormone-adjusted probiotic dosing, to optimize therapeutic outcomes in females with ASD.

## Methods

### Sample collection and gut microbiome isolation

The fecal samples from the typically developing (TD) individual were obtained as previously described (Liu et al., 2021). The fecal samples from the ASD individual used for gut microbial isolation were collected with approval from the Ethics Committee of Peking Union Medical College Hospital under the ethical approval number ZS-1393. The samples intended for bacterial isolation were kept fresh and transported to an anaerobic workstation (AW500, Electrotek, UK) within 2 hours of collection. The samples were suspended in anaerobic PBS and filtered through a 40 μm cell strainer. The resulting suspensions were divided into two portions: one portion was directly cultured on modified YCFA agar plates (YCFA agar medium supplemented with glucose at 1 g/L, hemin at 0.01 g/L, and L-cysteine at 1 g/L) and BHI agar plates supplemented with 5% defibrinated sheep blood. The other portion underwent alcohol pretreatment before being cultured on a different set of modified YCFA agar plates (YCFA agar medium supplemented with glucose at 1 g/L, hemin at 0.01 g/L, L-cysteine at 1 g/L, and sodium taurocholate at 0.1%) (Browne et al., 2016).

Colony isolation and identification were carried out as described in our previous study (Liu et al., 2021). All colonies on the plates were picked after cultivation periods of 24 h, 3 days, and 7 days, followed by incubation at 37°C under anaerobic conditions in the corresponding media for a duration of 1–7 days. Subsequently, 500 μl of the culture media were collected, and the bacterial pellets were harvested for PCR-based amplification of the 16S rRNA gene sequences using primers 27F and 1492R. The sequences of the PCR products were identified by aligning them against the NCBI 16S rRNA sequence database and the EZBioCloud database (updated on August 30, 2024). The threshold for 16S rRNA gene sequence identity for novel species was set at 98.7% (Liu et al., 2021). The phylogenetic tree of isolated gut microbiome strains was constructed by MEGA (Version 11.0.13) using the Neighbour-Joining Tree method. The 16S rRNA gene sequences of the isolated bacteria are provided in Supplementary Table 1. Isolated bacteria from the TD sample have been preserved in hGMB (Liu et al., 2021).

### Animals

All animal experiments complied with the National Institute of Health Guide for the Care and Use of Laboratory Animals. The permission for animal experiment procedures was granted by the Animal Ethics Committee at the Institute of Microbiology, Chinese Academy of Sciences.

Mice were maintained on a 12 h/12 h dark/light cycle (lights on at 7:00 am) in the animal facility at the Institute of Microbiology, Chinese Academy of Sciences, under specific pathogen-free conditions. Male and female BTBR *T*^+^ *Itpr3*^*tf*^/J (BTBR) mice purchased from Jackson Lab (America) were crossed to obtain BTBR mice offspring. After weaning, BTBR mice were reared separately by gender. C57BL/6J mice were purchased from SPF (Beijing) Biotechnology Co., Ltd (Beijing, China). After weaning, the mice were group-housed in cages containing two to five mice per cage. Mice from different experimental groups (C57BL/6J mice gavaged with PBS, BTBR mice gavaged with PBS, and BTBR mice gavaged with 4P-15) were housed separately. Male mice and samples from male mice were used in this study.

Animal numbers: The number of animals used in the behavioral tests (Figures 2b, c and Supplementary Figures 2a–c) were 10 for C57BL/6J mice gavaged with PBS, BTBR mice gavaged with PBS, and BTBR mice gavaged with 4P-15. The number of animals used for the cytokine analysis (Figure 3b) were 10, 8, and 10 for C57BL/6J mice gavaged with PBS, BTBR mice gavaged with PBS, and BTBR mice gavaged with 4P-15, respectively. The number of animals used in the 16S rRNA sequencing (Figures 4c, d and Supplementary Figures 3e–h) were 10 for C57BL/6J mice gavaged with PBS, BTBR mice gavaged with PBS, and BTBR mice gavaged with 4P-15. The number of animals used for intestinal indole detection (Figure 4b) were 10, 20, and 16 for C57BL/6J mice gavaged with PBS, BTBR mice gavaged with PBS, and BTBR mice gavaged with 4P-15, respectively. The number of animals used for the expression analysis of AhR, glutamate transporters and GABA receptors (Figures 5b–d) were 10 for C57BL/6J mice gavaged with PBS, BTBR mice gavaged with PBS, and BTBR mice gavaged with 4P-15. The number of animals used for neurotransmitter analysis (Supplementary Figure 2f) were 10 for BTBR mice gavaged with PBS and BTBR mice gavaged with 4P-15. The number of animals used for SCFA analysis (Supplementary Figure 2g) were 9 and 10 for BTBR mice gavaged with PBS and BTBR mice gavaged with 4P-15, respectively.

### *F. hominis* 4P-15 administration

*F. hominis* 4P-15 was grown anaerobically in a modified YCFA medium supplemented with 3 mM acetate (1M acetate-sodium acetate buffer) to stimulate bacterial growth (Duncan et al., 2002). The dosage of *F. hominis* 4P-15 chosen for colonization was based on previous publications (Yu et al., 2022b; Martín et al., 2015; Miquel et al., 2015), which reported effective dosages ranging from 1 × 10^8^ to 1 × 10^9^ colony-forming units (CFU). For each mouse in the *F. hominis* 4P-15 administration group, 4 × 10^8^ CFU were suspended in 200 μl PBS and delivered by gavage immediately. For the control group, an equal volume of PBS was given. The wean C57BL/6J or BTBR mice at 4 weeks old were gavaged with PBS or 4P-15 for a duration of 4 weeks, after which fecal samples were collected. The mice underwent behavioral tests at 8 weeks of age or were sacrificed for brain tissue collection. The 11-week-old mice that completed behavioral tests were sacrificed. The gavage regimen began when the mice were 4 weeks old and continued until they were sacrificed. The numbers of mice for gavage were 10, 20, and 20 for C57BL/6J mice gavaged with PBS, BTBR mice gavaged with PBS, and BTBR mice gavaged with 4P-15, respectively.

### Behavioral tests

The test mice were acclimated by handling them for three consecutive days before the experiments. Behavioral tests were carried out in the behavioral test room between 8:00 a.m. and 7:00 p.m., with the mice being transferred at least an hour in advance for acclimation (Yu et al., 2022b).

#### Self-grooming

The self-grooming test was conducted following the methodology outlined in a previous study (Gkogkas et al., 2013). Specifically, a test mouse was introduced into a clean arena and allowed to explore freely for 20 min. Self-grooming behavior (including genital or tail grooming, body grooming, paw or leg licking, and head washing) was then observed and recorded during the second 10-min interval, with the initial 10 min as the habituation phase.

#### Three-chamber social interaction test

The three-chamber sociability test was performed in a white plastic arena [60 cm × 15 cm] divided into three equal-sized chambers by clear movable plastic dividers. Three chambers were named ZONE 2, ZONE 1, and ZONE 3 from left to right. In the first session, the test mouse was introduced to the center of ZONE 1 and moved freely for 10 min. In the second session (social ability session), an age-, sex- and strain-matched stranger mouse (M1) previously habituated to the cylinder was placed in the center of ZONE 2, while an object in another cylinder (Object) was placed in the center of ZONE 3. The test mouse was then allowed to explore freely for 10 min. The movement was recorded by a camera over the arena. The duration time in ZONE 2 (M1) and ZONE 3 (Object) were analyzed using ANY-maze software. The percentage of duration time was calculated as duration time/600, and the preference index was calculated as (duration time in ZONE 2 – duration time in ZONE 3)/(duration time in ZONE 2 + duration time in ZONE 3). For the third session (social novelty preference session), another age-, sex- and strain-matched stranger mouse (M2) previously habituated to the cylinder was placed in the center of ZONE 3, while M1 was placed in the center of ZONE 2. The test mouse was then allowed to explore freely for 10 min. The movement was recorded by a camera over the arena. The duration time in ZONE 2 (M1) and ZONE 3 (M2) were analyzed using ANY-maze software. The percentage of duration time was calculated as duration time/600, and the preference index was calculated as (duration time in ZONE 3 – duration time in ZONE 2)/(duration time in ZONE 3 + duration time in ZONE 2) (Kaidanovich-Beilin et al., 2011; Yang et al., 2011).

#### Open-field test

The mouse was introduced to the center of the arena [40 cm (L) × 40 cm (W) × 50 cm (H)] and allowed to explore for 10 min. The video was recorded by a ceiling camera. Total distance traveled and time in the center (20 cm × 20 cm) were analyzed by Xeye Aba (3.2 version, Beijing Macroambition S&T Development, China) (Deng et al., 2019; Qiao et al., 2017).

#### Sucrose preference test

Mice were individually housed in standard cages for 72 h prior to experimentation to eliminate social interference. During the training phase, animals were acclimated to a two-bottle choice paradigm over two consecutive days: on Day 1, both bottles contained plain water, whereas on Day 2, both bottles were replaced with 1% (w/v) sucrose solution. Following a 24-h food deprivation period, the test session commenced with the simultaneous presentation of two identical bottles containing sucrose solution and plain water, respectively. To minimize position preference effects, bottle positions were systematically rotated every 12 h during the 24-h testing period. The weight of bottles was measured before and after the test, and the consumed weight was calculated. The preference index was calculated as sucrose solution consumed/(sucrose solution consumed + plain water consumed) (Yu et al., 2022a).

### Targeted metabolomics for indole and its derivatives

#### Sample preparation

Brain samples were taken out and subjected to rapid freezing in liquid nitrogen, subsequently stored at −80°C until later utilization. After thawing the the brain samples on ice, the cerebellum was isolated and homogenized in ice-cold methanol (MeOH) (5 μl/mg). After centrifugation at 14,000 rcf for 20 min at 4°C, 100 μl supernatant was transferred to a fresh tube and dried using a speed vacuum at room temperature. The pellets were dissolved in 10 μl of MeOH: H_2_O (1:1, v/v) and then subjected to centrifugation at 14,000 rcf for 20 min at 4°C. The supernatant was utilized for metabolomic analysis (Yu et al., 2022b).

Fecal samples were collected, immediately frozen in liquid nitrogen, and stored at −80°C until use. Frozen feces were thawed on ice and dispersed in ice-cold MeOH (5 μl/mg). The mixture was centrifuged at 14,000 rcf for 20 min at 4°C, and the supernatant was used for metabolomic assay (Yu et al., 2022b).

The fermentation broth samples of 4P-15 were collected after the strains had been grown anaerobically for 48 h and then centrifuged at 14,000 rcf for 20 min at 4°C. Three hundred microliter YCFA medium or supernatant of the broths were added to 1.5 ml ice-cold MeOH. The mixture was centrifuged at 14,000 rcf for 20 min at 4°C, and 1.2 ml of the supernatant was transferred to a new tube and then dried in a speed vacuum at room temperature. The pellets were re-suspended in 200 μl of MeOH: H_2_O (1:1, v/v) and then centrifuged at 14,000 rcf for 20 min at 4°C. The resulting supernatant was used for metabolomic analysis (Yu et al., 2022b).

#### Metabolomic assay

Metabolomics assays were conducted on a 6500 QTRAP triple quadrupole mass spectrometer (Applied Biosystems/Sciex) that was coupled to an ExionLC LC system (Applied Biosystems/Sciex). Chromatographic separations were accomplished on a Phenomenex Gemini^®^ 3 μm NX-C18 100 Å LC column (50 × 2 mm) (Yu et al., 2022b). Indole, indole-3-acetic acid, indole-3-aldehyde, indole-3-ethanol, indole-3-lactic acid, indole-3-propionic acid, tryptamine, kynurenine, and tryptophan were detected using multiple reaction monitoring modes with specific parent-daughter ion transitions at m/z ratios for each compound. Compounds were identified by comparing parent-daughter ion transitions and the retention time of reference standards: indole (118.0/91.0), indole-3-acetic acid (176.1/130.0), indole-3-aldehyde (146.1/91.0), indole-3-ethanol (162.1/144.1), indole-3-lactic acid (204.0/158.0), indole-3-propionic acid (190.0/130.1), and tryptamine (161.1/144.0) (Huang et al., 2021). The various concentrations of standards were employed for quantification purposes.

### Measurement of short-chain fatty acids (SCFAs)

For testing the SCFAs, including acetic acid, propionic acid, butyric acid, pentanoic acid, and valeric acid in feces, 0.1 g feces of each sample were freeze-dried and extracted in 1 ml methanol by 10-min ultrasound bathing in iced water. After the centrifuge, the supernatant liquid was prepared for GC-MS analysis. GC-MS analysis was performed on a GCMS-QP2010 Ultra with an autosampler (SHIMADZU) and the DB-wax capillary column (30 m, 0.25 mm i.d., 0.25 μm film thickness; SHIMADZU). The temperature of the oven was programmed from 80°C to 140°C at a 20°C/min gradient, with a 1 min hold; to 290°C at 3.5°C/min, with a 15 min hold. Injection of a 1 μl sample was performed at 280°C. The carrier gas, helium, flowed at 1.2 ml/min. The electronic impact was recorded at 70 eV. Referenced to standard curves, the products from feces were calculated by peak area (Li et al., 2022).

### Enzyme-linked immunosorbent assay (ELISA)

#### Neurotransmitters

The entire mouse brain including the cerebellum was homogenized by pulverization in cold PBS using a tissue homogenizer (60 Hz, 2 min, twice) at a ratio of 0.2 g brain tissue to 100 μl PBS and centrifuged at 3,000 rpm for 10 min. The supernatant was collected for ELISA using a Mouse 5-Hydroxytryptamine ELISA kit and a Mouse dopamine ELISA kit (QiSong, Beijing, China) according to the manufacturer's protocols.

#### Inflammatory cytokines

The right cerebral cortex of the brain was homogenized in lysis buffer containing a protease inhibitor cocktail (P0013B, Beyotime, China) and then centrifuged (4°C, 14,000 rcf, 20 min). The supernatant was utilized for ELISA analysis according to the manufacturer's guidelines. ELISA kits were used for IL-1β, IL-17A, TNFα, and IFNγ detection (Thermo Fisher, USA) and for IL-6 and IL-10 analysis (SinoBestBio, Shanghai, China). The concentrations of the inflammatory cytokines in the supernatant were normalized to the concentrations of total proteins (Yu et al., 2022b).

### 16S rRNA gene sequencing and data analysis

Genomic DNA was extracted from mouse cecal contents or human feces by QIAamp PowerFecal^®^ Pro DNA kit (Qiagen, United States). The V3-V4 region of the 16S rRNA gene was amplified using the primer set 338F (5′-CCTACGGGNGGCWGCAG-3′) and 806R (5′-GACTACHVGGGTWTCTAAT-3′). PCR reactions were amplified using PremixTaq (TaKaRa, China), DNA products were purified by gel electrophoresis, and a sequencing adapter was connected to build the library followed by the standard protocol of NEBNext^®^UltraTM DNALibraryPrepKitforIllumina^®^. The 16S rRNA gene amplicon sequencing was conducted on Illumina novaseq 6000 PE250 platform.

The raw sequencing data of 16S rRNA gene sequences were processed using QIIME2 (v2021.4.0) (Bolyen et al., 2019). Sequences were trimmed to remove primer and adapter sequences. The trimmed raw reads were then analyzed with DADA2 (Callahan et al., 2016) to perform quality trimming, denoising, error-correction, paired-end read merging, chimera removal, and dereplication. The produced amplicon sequence variants (ASVs) were taxonomically classified according to the SILVA database (release 138) using “classify-sklearn,” after training the classifier on the S database (v.2022.10) (McDonald et al., 2024) with 341F/806R primers. Subsequently, the microbial profile table was exported for downstream analyses.

For diversity analysis, the microbial table was first normalized by total sum reads. Alpha diversity was assessed using the “diversity” function in the R package vegan, with statistical significance determined by the “aov” function. Then the “p.adjust” function (method = “bonferroni”) in R. Beta diversity analysis involved conducting principal component analysis (PCA) using the “prcomp” function (center = T, scale = T) in R and visualization with the ggbioplot R package. Multivariate analysis of variance in beta diversity analysis using distance matrices (PERMANOVA) was carried out with the “adonis2” function (permutations = 9999, method = “euclidean”) in the vegan package.

The metabolic gene content was estimated from 16S rRNA gene data using Phylogenetic Investigation of Communities by Reconstruction of Unobserved States 2 (PICRUSt2) pipeline with default parameters. ASVs with nearest-sequenced taxon index (NSTI) values >2 were filtered from the analysis. KEGG orthology (KO), and enzyme Commission number (EC number) metagenomes, and MetaCyc pathway abundances were predicted with PICRUSt2 (Renga et al., 2023).

### qPCR analysis

Total RNA extracted from the left cerebral cortex of mice was used for cDNA preparation using a Hifair^®^ AdvanceFast One-step RT-gDNA Digestion SuperMix for qPCR (11151ES60, Yeasen, Shanghai, China). Real-time PCR analysis was performed using Hieff^®^ qPCR SYBR Green Master Mix (11203ES08, Yeasen, Shanghai, China) to determine the expression levels of glutamate transporters (*Slc1a1, Slc1a2, Slc1a3*), GABA receptors (*Gabra1, Gabrb2, Gabrg2, Gabbr1*), AhR (*Ahr*), cytochrome P450 (CYP) enzymes (*Cyp1b1*), and the *Gapdh* gene. The sequences of primers were provided in Supplementary Table 2.

### Statistical analysis

False discovery rate (FDR)-adjusted *P*-values determined by two-sided Wilcoxon rank-sum tests was used for analyzing *Feacalibacterium* abundance differences in published cohorts (Figure 1a and Supplementary Figure 1). Mann-Whitney (Supplementary Figures 2b, c, f, g) and Student's *t*-tests (Figure 5a and Supplementary Figures 3b–d) were used for comparing data between two groups. One-way ANOVA with Tukey's multiple comparison tests [Figures 2b, c (right panel), Figures 3b, 5b–d] and one-way ANOVA with multiple comparisons by controlling FDR (Figures 4b, d) were used for multiple comparisons across three groups. Two-way ANOVA with Tukey's multiple comparison tests were used for comparing the effects of two factors on a response variable [Figure 2c (lefe panel) and Supplementary Figure 2a (left panel)]. Spearman correlation tests (Figure 5e and Supplementary Figure 4b) and Pearson correlation tests (Supplementary Figures 2d, e) in R were used to quantify associations. PERMANOVA with the adonis2 function (vegan package) using Euclidean distances was used for beta diversity analysis (Figure 4c).

## Data Availability

The assembled genome of 4P-15 was available at NODE with the project accession OEP001106 (https://www.biosino.org/node/project/detail/OEP001106) (Liu et al., 2021). The 16S rRNA gene sequence data have been deposited in the the Genome Sequence Archive (GSA) in National Genomics Data Center, Beijing Institute of Genomics (China National Center for Bioinformation), Chinese Academy of Sciences under the accession code PRJCA038009 (https://ngdc.cncb.ac.cn/search/specific?db=biosample&q=PRJCA038009).
