# Supplementary material for: The gut commensal Faecalibacterium hominis attenuates indole—AhR signaling and restores ASD—like behaviors with BTBR mice
Source: Front Microbiol. 2025 Aug 22;16:1640149. doi: 10.3389/fmicb.2025.1640149 (PMC12411466; doi:10.3389/fmicb.2025.1640149)
Supplement: Supplementary file 1 [file Supplementary_file_1.docx]

**Supplemental Information**

**Supplementary Fig. 1 | The potential ASD mitigating effect of *Faecalibacterium*.** The relative abundance of *Faecalibacterium* in male and female individuals between TD and ASD. The figures were based on the original data of a selected ASD cohort (ID number 11169 in the database “Qiita”)[17]. Quantitative data are shown as the mean ± SEM. The *P* value was determined by the two-sided Wilcoxon rank-sum test and the adjusted *P* value was evaluated using the false discovery rate (FDR) correction of *P* value, and the significance was indicated by adjusted *P* value.

**Supplementary Fig. 2 | 4P-15 restored the core ASD-like behaviors of BTBR mice. a**, Percentage of duration time (left panel) and the preference index (right panel) in the social novelty preference session of the three-chamber social interaction test. n = 10 mice for each group. **b**, Percentage of time in the center in the open-field test. n = 10 mice for each group. **c**, Sucrose preference in the sucrose preference test. n = 10 mice for each group. **d**, Scatter plots showing the association between the log values of 4P-15 copy number (X-axis) and the stereotyped behavior indicated by grooming time in the self-grooming test (Y-axis) in BTBR mice gavaged with 4P-15. n = 3 mice for each group. Statistical significances (*P* value) and correlation coefficients (R^2^) were determined by the function *cor.test* (method = "pearson") in R. The significant difference was defined as the *P* value < 0.05. **e**, Scatter plots showing the association between the log values of 4P-15 copy number (X-axis) and the impaired social communication indicated by the duration time in ZONE 3 (Object) in the social ability session of the three-chamber social interaction test (Y-axis) in BTBR mice gavaged with 4P-15. n = 3 mice for each group. Statistical significances (*P* value) and correlation coefficients (R^2^) were determined by the function *cor.test* (method = "pearson") in R. The significant difference was defined as the *P* value < 0.05. **f**, The concentrations of neurotransmitters in the brain. n = 10 mice for each group. **g**, The concentrations of SCFAs in the feces. n = 9 and 10 mice, respectively.

Quantitative data are shown as the mean ± SEM. Statistical analysis was determined by the two-way ANOVA with two-tailed Turkey’s test for multiple comparisons (**a** (left panel)), one-way ANOVA with two-tailed Tukey’s multiple comparison test (**a** (right panel)) and two-tailed Mann-Whitney test (**b, c, f** and **g**). Significance was indicated by *P* value (**b-g**) and adjusted *P* value (**a**).

**Supplementary Fig. 3 | 4P-15 modulates intestinal indole and its derivatives as well as gut microbes in BTBR mice. a**, The indole biodegradation and biotransformation pathways and related enzymes[32]. **b**, The IAA concentrations in the fermentation broth samples of 4P-15 compared with its YCFA medium. **c**, The indole concentrations in the fermentation broth samples of 4P-15 compared with its YCFA medium supplemented with 1 μg/ml indole. **d**, The IPA concentrations in the fermentation broth samples of 4P-15 compared with its YCFA medium supplemented with 1 μg/ml IPA. **e**-**h**, A 16s rRNA gene sequencing analysis of gut microbiota from C57BL/6J mice treated with PBS, BTBR mice treated with PBS, and BTBR mice treated with 4P-15. n = 10 mice for each group. **e,** The Shannon and Simpson index for alpha diversity at the ASV level. **f,** PCA plots of the fecal microbiota composition at the genus level (PERMANOVA). **g**, The relative abundance of *Bacteroides* and *Lactobacillus*. **h**, The gene content of tryptophanase (EC:4.1.99.1) predicted by PICRUSt2 from 16S rRNA gene sequencing data. Quantitative data are shown as the mean ± SEM. The *P* value and adjusted *P* value were determined by the two-sided Student’s *t*-test and the one-way ANOVA with FDR correction (**e**, **g**, and **h**), respectively. Significance was indicated by *P* value (**b**-**d**) and adjusted *P* value (**e**, **g**, and **h**).

**Supplementary Fig. 4 | *F. hominis* 4P-15 modulates indoles and AhR signaling in the brain of BTBR mice. a**, qPCR analysis showing the expression of *Cyp1b1* in the cerebral cortex of C57BL/6J mice gavaged with PBS, BTBR mice with PBS, and BTBR mice with 4P-15. Quantitative data are shown as the mean ± SEM. Statistical analysis was determined by the one-tailed Mann-Whitney test. Significance was indicated by *P* value. **b**, Scatter plots showing the association between the mRNA levels of AhR downstream genes (X-axis) and the concentrations of AhR ligands (Y-axis) in BTBR mice gavaged with 4P-15. n = 8 mice for each group. Statistical significances (*P* value) and correlation coefficients (R^2^) were determined by the function *cor.test* (method = "spearman") in R. The significant difference was defined as the *P* value < 0.05. Significance was indicated by *P* value.
